# In this month’s Bulletin

**DOI:** 10.2471/BLT.18.001218

**Published:** 2018-12-01

In the editorial section, Robert Marten et al. (798) discuss a shift in global governance needed to reach the sustainable development goals. Shamsuzzoha B Sayeed et al. (799) describe national efforts to integrate health-care quality improvement initiatives.

In the news section, Vijay Shankar Balakrishnan (802–803) reports the challenges in preventing, detecting and treating hepatitis B and C infections in the European Region. Jean-Jacques Muyembe Tamfum talks to Fiona Fleck (804–805) about his decades of experience in responding to Ebola virus disease outbreaks.

## China

### Improving urban healthcare 

Xin Wang et al. (843–852) describe moves toward people-centred integrated care.

## Japan

### Living past retirement

Shohei Okamoto et al. (826–833) estimate the health effects for men of working past their retirement age.

## Malawi 

### When the last mile is on water 

Francesco Grandesso et al. (817–825) document a cholera vaccination programme. 

## South Africa

### Using the *International Statistical Classification of Diseases*

Tina Lavin et al. (806–816) classify perinatal mortality data.

## Thailand

### Surviving snakebite

Netnapis Suchonwanich & Winai Wananukul (853–857) document improved access to antidotes and antivenoms.

## Zimbabwe 

### Delivering services

Anna Hidle et al. (834–842) count the costs of a human papillomavirus vaccination project.

## Global

### What happened to the environment?

Graziella Iossa & Piran CL White (858–860) identify a missing link in plans to combat antimicrobial resistance.

### Continuous, permanent, compulsory

Debra Jackson et al. (861–863) explain how civil registration and vital statistics are used by health systems.

